# *Notes from the Field:* Universal Statewide Laboratory Testing for SARS-CoV-2 in Nursing Homes — West Virginia, April 21–May 8, 2020

**DOI:** 10.15585/mmwr.mm6934a4

**Published:** 2020-08-28

**Authors:** Shannon M. McBee, Erica D. Thomasson, Melissa A. Scott, Christy L. Reed, Lauren Epstein, Amy Atkins, Catherine C. Slemp

**Affiliations:** ^1^West Virginia Bureau for Public Health, West Virginia Department of Health and Human Resources, Charleston, West Virginia; ^2^Division of State and Local Readiness, Center for Preparedness and Response, CDC; ^3^Division of Healthcare Quality Promotion, National Center for Emerging and Zoonotic Infectious Diseases, CDC.

Outbreaks of coronavirus disease 2019 (COVID-19) in nursing homes can severely affect older adults. During March 17–April 16, 2020, seven nursing homes in West Virginia reported 307 COVID-19 cases among both residents and staff members; four of the nursing homes reported outbreaks involving 20–40 residents. On April 17, the governor of West Virginia issued Executive Order 27–20* directing the West Virginia Bureau for Public Health (WVBPH) to coordinate universal testing for SARS-CoV-2, the virus that causes COVID-19, among residents and staff members of all 123 West Virginia nursing homes, irrespective of symptoms. During April 21–May 8, universal testing was conducted in all 123 West Virginia nursing homes, with 42 COVID-19 cases identified in 28 (23%) nursing homes; the 42 cases occurred in 11 residents (0.1% of residents tested) and 31 staff members (0.2%).

Beginning April 21, nasopharyngeal or nasal swabs were collected from residents by in-house staff members, local health departments, or the West Virginia National Guard. Specimens were tested at a private laboratory using a statewide contract or at other commercial laboratories arranged by the nursing homes, using real-time reverse transcription–polymerase chain reaction. In nursing homes with active outbreaks, all persons received testing who had previously tested negative or had not been tested. An outbreak was defined as the detection of two or more laboratory-confirmed COVID-19 cases within 14 days among staff members or residents in a nursing home. All residents with positive SARS-CoV-2 test results were isolated in private rooms, and transmission-based precautions were implemented by WVBPH in alignment with CDC guidance.^†^ Health care workers with positive test results were required to isolate at home until they met the criteria to discontinue home isolation following CDC guidance and were monitored by public health officials through daily text messaging. Health care workers with negative test results who were close contacts of residents or other staff members with confirmed COVID-19 were instructed to quarantine at home for 14 days from their last exposure. Following universal testing, nursing homes screened staff members and residents daily and tested anyone with signs or symptoms of COVID-19. If additional cases were identified, testing was also performed for close contacts of patients, including all residents cared for by the same health care worker. WVBPH monitored nursing homes’ adherence to infection prevention and control measures through conference calls, and facilities twice weekly submitted line lists of residents and staff members who were symptomatic or who had a positive SARS-CoV-2 test result.

During April 21–May 8, universal testing was conducted in all 123 West Virginia nursing homes. Receiving testing was declined by 1.3% (115 of 9,026) of residents and 1.7% (239 of 13,926) of staff members. Among the 8,911 residents and 13,687 staff members who were tested, 42 COVID-19 cases were identified in 28 (23%) nursing homes, none of which had previously experienced an outbreak. The 42 cases occurred in 11 residents (0.1% of residents tested) and 31 staff members (0.2% of tested staff members). The 42 identified cases represented 20 single cases from 20 facilities and 22 outbreak-associated cases, representing new outbreaks (ranging in size from two to six persons) in eight facilities (Table). The prevalence of positive SARS-CoV-2 test results was lower in nursing homes with COVID-19 outbreaks during universal testing (0.9% of residents and 1.9% of staff members) than it was during earlier outbreaks when testing was triggered by daily symptom-based resident screening (38.1%) and preshift employee screening (16.3%). Before universal testing, 32 COVID-19–associated nursing home deaths had been reported; however, no deaths occurred among residents with COVID-19 who were identified during universal testing.

**TABLE:** Characteristics of COVID-19 outbreaks in nursing homes before and during implementation of universal testing* of all residents and staff members — West Virginia, March 17–May 8, 2020

Characteristic	No. (%)^†^					
	Total at nursing home	Tested	Refused	Positive results	Asymptomatic	Deaths
**Outbreaks identified before implementation of universal testing (March 17–April 16, 2020) (N = 7)**						
Staff members	793 (100)	736 (92.8)	57 (7.2)	129 (16.3)	29 (22.5)	0 (0.0)
Residents	467 (100)	463 (99.2)	4 (0.9)	178 (38.1)	129 (72.5)	32 (18.0)
Total	1,260 (100)	1,199 (95.2)	61 (4.8)	307 (24.4)	158 (51.5)	32 (10.4)
**Outbreaks identified during implementation of universal testing^§^ (April 21–May 8, 2020) (N = 8)**						
**Outbreak 1**						
Staff members	80 (100)	80 (100)	0 (0.0)	2 (2.5)	0 (0.0)	0 (0.0)
Residents	54 (100)	54 (100)	0 (0.0)	0 (0.0)	0 (0.0)	0 (0.0)
**Outbreak 2**						
Staff members	70 (100)	70 (100)	0 (0.0)	2 (2.9)	0 (0.0)	0 (0.0)
Residents	58 (100)	58 (100)	0 (0.0)	0 (0.0)	0 (0.0)	0 (0.0)
**Outbreak 3**						
Staff members	110 (100)	110 (100)	0 (0.0)	0 (0.0)	0 (0.0)	0 (0.0)
Residents	72 (100)	72 (100)	0 (0.0)	2 (2.8)	0 (0.0)	0 (0.0)
**Outbreak 4**						
Staff members	110 (100)	110 (100)	0 (0.0)	3 (2.7)	0 (0.0)	0 (0.0)
Residents	46 (100)	46 (100)	0 (0.0)	0 (0.0)	0 (0.0)	0 (0.0)
**Outbreak 5**						
Staff members	106 (100)	106 (100)	0 (0.0)	1 (0.9)	0 (0.0)	0 (0.0)
Residents	49 (100)	49 (100)	0 (0.0)	1 (2.0)	0 (0.0)	0 (0.0)
**Outbreak 6**						
Staff members	107 (100)	107 (100)	0 (0.0)	2 (1.9)	0 (0.0)	0 (0.0)
Residents	43 (100)	43 (100)	0 (0.0)	0 (0.0)	0 (0.0)	0 (0.0)
**Outbreak 7**						
Staff members	163 (100)	163 (100)	0 (0.0)	5 (3.0)	5 (100)	0 (0.0)
Residents	121 (100)	121 (100)	0 (0.0)	1 (0.8)	1 (100)	0 (0.0)
**Outbreak 8**						
Staff members	157 (100)	157 (100)	0 (0.0)	2 (1.3)	1 (50.0)	0 (0.0)
Residents	108 (100)	107 (100)	1 (0.9)	1 (0.9)	1 (100)	0 (0.0)
**All 8 outbreaks**						
Staff members	903 (100)	903 (100)	0 (0.0)	17 (1.9)	6 (35.2)	0 (0.0)
Residents	551 (100)	550 (99.8)	1 (0.2)	5 (0.9)	2 (40.0)	0 (0.0)
**Total**	**1,454 (100)**	**1453 (99.9)**	**1 (0.0)**	**22 (1.5)**	**8 (36.4)**	**0 (0.0)**

**Abbreviation**: COVID-19 = coronavirus disease 2019.

* Universal testing was defined as facility-wide viral testing of all residents and staff members in a nursing home, irrespective of symptoms; Executive Order 27–20 (https://governor.wv.gov/Documents/2020%20Executive%20Orders/Executive-Order-April-17-2020-Nursing-Home-Testing.pdf) was issued on April 17, 2020, directing SARS-CoV-2 testing in all West Virginia nursing homes.

^†^ Percentages tested, refused, and positive are percentages of total; percentage asymptomatic and percentage of deaths are percentages of persons with a positive test result.

^§^ 20 additional isolated (nonoutbreak–associated) COVID-19 cases were identified at 20 nursing homes during universal testing.

In six of the eight nursing homes with newly identified COVID-19 outbreaks where cohorting of residents with positive SARS-CoV-2 test results and exclusion of staff members with positive test results were implemented, daily follow-up symptom screening of all residents and staff members for 28 days (the upper bound of two incubation periods) found that further transmission did not occur. Two facilities experienced minimal transmission beyond the initial cases detected during universal testing.

Universal testing identified eight outbreaks with 17 staff members and five residents who tested positive for SARS-CoV-2, including six staff members and two residents who were asymptomatic (Table). The testing likely prevented the occurrence of ongoing transmission and larger outbreaks, had the asymptomatic infections gone undetected. Proactive universal testing prevented additional infections, as illustrated by the lower percentages of residents and staff members with positive test results in outbreaks identified through universal testing compared with those identified through symptom screening. Universal testing helped estimate the prevalence of COVID-19 in a population at increased risk for serious COVID-19 outcomes (*1*) so that public health resources could be allocated to prevent further spread (*2*). Statewide universal testing enabled rapid implementation of infection prevention and control measures that likely prevented the occurrence of larger outbreaks. Since completing universal screening, West Virginia has maintained symptom screening in nursing homes, revised its outbreak case definition to constitute a single case in a nursing home, and adopted universal testing of all residents and staff members in response to an outbreak with weekly testing for a period of at least 14 days since the most recent positive result.

For the period May 8–July 26, following completion of universal testing and under the new procedures, 18 COVID-19 outbreaks were identified in West Virginia nursing homes, 12 of which involved five or fewer cases. Although universal testing is resource-intensive, it has proven essential to limiting COVID-19 transmission in nursing homes and has reduced the impact of the pandemic on this vulnerable population in West Virginia.

## Footnotes

*
https://governor.wv.gov/Documents/2020%20Executive%20Orders/Executive-Order-April-17-2020-Nursing-Home-Testing.pdf .

†
https://www.cdc.gov/coronavirus/2019-ncov/hcp/nursing-homes-responding.html .
